# Pathobiology of cancer metastasis: a short account

**DOI:** 10.1186/1475-2867-12-24

**Published:** 2012-06-07

**Authors:** Liviu Feller, Beverley Kramer, Johan Lemmer

**Affiliations:** 1 Department of Periodontology and Oral Medicine, School of Oral Health Sciences, Faculty of Health Sciences, University of Limpopo, Medunsa Campus, Medunsa, 0204, South Africa; 2 School of Anatomical Sciences, Faculty of Health Sciences, University of Witwatersrand, Johannesburg, South Africa; 3 Department of Periodontology and Oral Medicine, University of Limpopo, Medunsa campus, South Africa; Professor Emeritus of Oral Medicine and Periodontology, University of Witwatersrand, Johannesburg, South Africa

**Keywords:** Cancer stem cells, Pre-metastatic niche

## Abstract

Cancer-initiating cells display aberrant functional and phenotypic characteristics of normal stem cells from which they evolved by accumulation of multiple cytogenetic and/or epigenetic alterations. Signal transduction pathways which are essential for normal stem cell function are abnormally expressed by cancer cells, with a cancer cell phenotype playing an essential role in cancerization and metastasis.

Local tumour progression, metastasis and metastatic tumour growth are mediated by direct cell-to-cell and paracrine reciprocal interactions between cancer cells and various stromal cells including fibroblasts, macrophages, bone marrow derived stem cells and progenitor cells. These interactions mediate breakdown of basement membrane barriers and angiogenesis both locally at the invasive front of the primary tumour and at the distant metastatic site; attract primary tumour cells to the candidate metastatic site; and promote proliferation, survival and growth of primary tumour cells and of metastatic cells at their distant site.

It is the purpose of this article to highlight the analogies between some of the genetic programs of normal stem cells, and of cancer cells participating in the process of metastasis.

## Background

A primary malignant tumour consists of a heterogeneous population of cancer cells comprising stem/progenitor cells, cells at different stages of differentiation and de-differentiated cells [1]. Cancer stem cells and de-differentiated cancer cells are capable of using several intracellular pathways that are analogous to those used by normal stem/progenitor cells and their progeny during development, despite the dysregulation of many of their biological functions. Most probably the precursor cancer cells originate either as tissue specific stem cells, or as progenitor cells that have undergone cancerous transformation expressing dysregulated cellular signalling pathways that in normal cells would be strictly regulated [2]. As cancer stem cells are long-lived and possess persistent self-renewal capabilities, they are well endowed to initiate metastatic cancers [2-4].

During the process of de-differentiation, there is de-activation of repressive mechanisms of developmental transcription factors, and activation of dormant intracellular signalling pathways which are ordinarily expressed during development by normal stem cells [1-6].

Stem cells that initiate cancer display several properties that normal stem cells exhibit, including the capability for self–renewal and differentiation, active expression of telomerase, motility, and the ability to migrate. Furthermore, metastatic cells like normal stem cells, function and number of which are regulated by signals from the niche in the microenvironment in which they are located, require a specific microenvironmental niche at their destination site in order to initiate and sustain metastatic growth [1,2,7].

It is the purpose of this article to discuss some of the cellular genetic programs analogous to those that are used by tissue specific stem cells, employed by cancer cells in the process of metastasis.

### The metastatic process

In general, the process of metastasis comprises an orderly sequence of pathobiological events starting with local invasion by tumour cells, continuing with intravasation of the cells and their survival in the bloodstream, and ending with their extravasation, and ultimately with infiltration and colonization of distant tissues [1,8]. These events are driven by gene products of metastatic cells, and by direct cell-to-cell and paracrine interactions between cancer cells and various stromal cells, both in the primary and in the metastatic tumour microenvironments [8-15].

Although it is evident that the cancer-associated reactive stroma surrounding the primary tumour promotes tumour growth and invasiveness, it is not clear whether signals from the microenvironment induce genotypic and phenotypic changes in the cancer cells without which metastasis cannot occur [16]; or whether these genotypic and phenotypic properties are induced by inherent altered intracellular transduction pathways and gene expression, that have been present from the cancer stem cell stage.

During disease progression, cancer cells activate local stromal cells including resident fibroblasts and macrophages, and attract to the primary tumour circulating monocytes and platelets. In turn, the reactive stroma-associated cytokines, chemokines, growth factors and matrix metalloproteinases mediate attraction of bone marrow-derived stem and progenitor cells to the microenvironment of the primary tumour. These active agents also mediate angiogenesis, degradation of basement membrane barriers and other extracellular matrix components, as well as detachment, motility and migration of cells from the primary cancer, thus promoting local tumour growth and invasion [8-12,16,17].

### The pre-metastatic niche

The destination and growth of cells metastasising from the primary cancer, are dictated by specific metastatic genes expressed by the disseminating cells, by the characteristics of the microenvironment of the candidate metastatic sites, and by the interaction between the stromal microenvironment at these sites and the metastatic cells [8,10-12,18,19].

Primary cancer cells and their associated reactive stromal cells secrete humoral factors including an array of cytokines, chemokines and growth factors which determine tropism for specific distant tissues, and promote metastatic colonization and infiltration [10,18,19]. It is probable that similar humoral factors released by different types of primary cancers, will result in those different cancers metastasising to the same distant tissues; whereas other humoral factors released by a primary cancer will account for its tendency to metastasise to particular tissues or organs [18]. Thus, tumour-specific biologically active factors will dictate the pattern of cancer metastasis [19].

These biological factors, including vascular endothelial growth factor and placental growth factor activate fibroblasts in the stromal microenvironment of the candidate metastatic sites resulting specifically in enhanced fibronectin expression. Concurrently with this process, these and other biological factors recruit haematopoietic progenitor cells (HPCs) from the bone marrow to the fibronectin rich stroma. The fibronectin provides a directional signal to the arriving HPCs, and serves as an adhesive substrate in which HPCs aggregate in clusters. Subsequently, the clusters of HPCs secrete chemokines, cytokines, growth factors and adhesion molecules required for the chemoattraction of primary cancer cells, and for the creation of a favourable microenvironment called a pre-metastatic niche. This niche will be colonized by metastatic cells [10,18,19].

Soon after these pathobiological events, HPCs in the niche now colonized by metastatic cells recruit bone-marrow derived endothelial progenitor cells (EPC) that will contribute to the vascularisation of the site, promoting growth of micrometastases [18,19].

Thus, the changes in the microenvironment of a candidate metastatic site mediated and aided by products of the primary cancer-associated stromal cells, by fibronectin, by HPCs, and by EPCs, create a supportive microenvironment which promotes recruitment, attachment, survival and growth of the metastasised cells [19].

The ability of cancer stem cells to induce angiogenesis, to migrate, to invade tissues and blood vessels and to infiltrate and colonize distant tissues is in part mediated by cellular pathways which are expressed ordinarily by normal tissue-specific stem/progenitor cells. Three of these signalling pathways which are particularly important in carcinogenesis are the interaction between chemokine stromal derived factor-1 (SDF-1) and its chemokine receptor CXCR4, the epithelial to mesenchymal transition (EMT) pathway and the Wnt pathway [4,5,8,10-12,16,17,20-30].

### The SDF-1-CXCR4 axis

The SDF-1-CXCR4 axis is essential in the function of normal stem/progenitor cells; and in carcinogenesis it mediates the growth of primary cancers and the development of cancer metastases [20]. CXCR4 receptors are expressed by a variety of tissue-specific stem cells and by several types of cancer cells, and in fact, all cancer cells that metastasise to bone express CXCR4 receptors [14,17,26,31].

SDF-1 is produced by stromal cells of mesenchymal origin including fibroblasts, osteoblasts and endothelial cells, and by haematopoietic stem/progenitor cells and endothelial stem/progenitor cells of bone marrow [20,26]. SDF-1 is highly expressed in lymphatic tissue, lung, liver and bone [20]. The expression of both SDF-1 and CXCR4 is upregulated by biological factors associated with tissue damage or tissue hypoxia. A prominent promoter of SDF-1-CXCR4 expression is hypoxia-inducible factor-1 (HIF-1) which is upregulated by hypoxia within the progressively growing primary cancer [11,17,20].

Both tissue-specific cancer stem/progenitor cells and their normal counterparts, express CXCR4 receptors and respond to chemoattractant signals generated by SDF-1, resulting in their directional migration along the SDF-1 concentration gradient. The stem/progenitor cells will consequently arrive at and be retained in the microenvironment which expresses high levels of SDF-1 [8,11,20].

In response to stimulation by SDF-1, CXCR4-positive cells display motility and increased adhesion properties, and secrete angiogenic factors (e.g. VEGF) and matrix metalloproteinases [10,20]. Furthermore SDF-1 may promote survival and proliferation of cancer cells expressing CXCR4 receptors [17].

Upon exposure to SDF-1, cancer cells expressing CXCR4 receptors display rearrangement of cytoskeletal proteins. This promotes cell motility leading to migration along the SDF-1 gradient, resulting in local invasion by primary tumour cells. SDF-1 also modulates adhesion of CXCR4-positive cancer cells to endothelial cells, to fibrinogen and to fibronectin by activating specific integrins and other cell surface molecules including VCAM-1, ICAM-1 on endothelial cells. This facilitates migration through the endothelial cell layer [17,20].

The transendothelial migration is further facilitated by SDF-1 induced secretion of matrix metalloproteinases (MMP)-2 and MMP-9 by CXCR4 positive cancer cells. The increased expression of MMP’s leads to degradation of extracellular matrix and of components of basement membrane increasing vascular permeability and cancer cell extravasation, and facilitating the locomotion of cancer cells and their invasion of tissues [17,32].

SDF-1 either alone or in combination with other factors mediates the trafficking and homing of tissue specific cancer stem/progenitor cells to cell-specific niches in the target tissue. SDF-1 also mediates trafficking of bone marrow derived haematopoietic progenitor cells to the metastatic niche, facilitating angiogenesis and the formation of a favourable microenvironment that is critical for metastatic growth [10,17,18].

### Epithelial-mesenchymal transition

Epithelial-mesenchymal transition (EMT) is a cellular genetic program expressed during embryogenesis that governs morphogenesis. EMT transcription factors mediate the conversion of polarized immotile epithelial cells to motile mesenchymal progenitor cells [27,28].

Molecular changes occur in the transition from an epithelial to a mesenchymal phenotype. For example, EMT transcription factors suppress E-cadherin expression in epithelial cells resulting in functional loss of cell-to-cell adhesion. They also trigger complex changes in cellular architecture such as alterations in actin cytoskeleton resulting in the acquisition of a mesenchymal phenotype capable of motility and migration [27].

Dysregulation of the EMT programme contributes to tumour initiation, invasion and metastatic spread. Signalling pathways which induce EMT transitions include TGF-β, Notch, Wnt, and Hedgehog, and these in turn act via transcription factors such as Twist1, Twist2, Snail, Slug, ZEB1 and ZEB2 [33]. Common pathways in EMT have been identified in development and in oncogenesis/cancerization [33]. These commonalities suggest that dormant developmental pathways are triggered in cancers and contribute to tumour progression. For example, inhibition of Wnt signalling has been shown to reduce the capacity of cancer cells to self-renewal and blocked tumour formation by repressing EMT transcription factors [34].

Thus, during cancerization, the EMT genetic program may be reactivated in de-differentiated cancer cells [21], or be aberrantly expressed by cancer stem cells [29]. Epidermal growth factor, transforming growth factor-β, fibroblast growth factor and hypoxia-inducible factor 1α (HIF1α) released in the microenvironment of the primary cancer are the biological agents that have the capacity to induce EMT in cancer cells [10,16,28]. Cancer cells which undergo EMT are predominantly found at the invasive front of the primary tumour [35].

Similarly to what occurs during embryogenesis, during the metastatic cascade, cancer stem cells or de-differentiated cancer cells of epithelial origin which express the EMT transcription factor Twist, promote loss of E-cadherin-mediated cell-to-cell adhesion and cell motility with subsequent expression of mesenchymal genes. Cancer cells undergoing EMT display fibroblast-like spindle morphology and express fibronectin, vimentin, smooth muscle actin, and N-cadherin [21]. Furthermore, transcription factors associated with EMT such as Twist or Snail, also mediate the proliferation of cancer stem cells with self-renewal capacity, thus increasing the number of cancer initiating cells [29], and mediate cancer stem cell survival [36]. These properties of increased proliferation and prolonged survival greatly contribute to the successful colonization of disseminating cancer cells at a metastatic site [29].

EMT may be a transient phenomemon, so that after initial dissemination most metastatic cells which had undergone EMT, revert via a mesenchymal-epithelial transition program, re-acquiring an epithelial phenotype at their metastatic destination [28,35].

### Wnt signalling pathways

Wnt signalling transduction pathways mediate developmental processes during embryogenesis by maintaining the integrity of the stem cell niche, and by regulating stem cell division, proliferation and migration, and tissue polarity [23,37]. Through the activation of the EMT genetic program, Wnt signalling is involved in gastrulation and in the development of the heart and the neural crest [28,35].

One of the several Wnt signalling pathways, the canonical pathway, is associated with translocation of β-catenin from adherence junctions to the nucleus, with subsequent activation of β-catenin/T cell factor (TCF) transcriptional complexes. The mobilization of β-catenin from the adherence junctions with the subsequent functional loss of E-cadherin, together with β-catenin/TCF direct activation of E-cadherin repressor SLUG, may bring about EMT [35,37].

The activation of the canonical Wnt pathway occurs when Wnt ligands concurrently engage with Wnt receptor Frizzled and with the low-density lipoprotein receptor-related protein (LRP) 5/6 [23,24]. The Wnt canonical signalling is regulated by a number of antagonists, including Dikkopf 1 (DKK1), a soluble secreted protein. The interaction between DKK1 and LRP 5/6 brings about active inhibition of the Wnt/β-catenin signalling pathway [23,24].

During carcinogenesis Wnt signalling is aberrantly activated in cancer stem cells and de-differentiated cancer cells contributing to tumour invasion and metastasis. Dysregulated expression of Wnt signalling increases the proliferation of cancer stem cells and promote their resistance to apoptosis [3,35,37]. The dysfunctional Wnt signalling starts early in carcinogenesis and is maintained throughout the course of tumour progression. However, Wnt-associated activation of EMT that results in cancer cell detachment, motility and migration with subsequent invasion and metastasis, is a relatively late event [35].

Wnt proteins (ligands, receptors and co-receptors) in differentiated osteoblasts promote expression of genes associated with bone formation, and indirectly control osteoclast differentiation and function by regulating the secretion of osteoprotegerin by osteoblasts, thus playing an important role in regulating bone mass [23,24].

In metastatic bone disease, the metastatic cells may either activate the Wnt signalling pathway or down-regulate the inhibitory functions of DKK-1 on the Wnt signalling, resulting in bone formation and in the formation of an osteoblastic lesion; or the metastatic cells may inhibit the Wnt signalling through upregulation of DKK-1, leading to the inhibition of osteoblastic functions with consequent development of osteolytic lesions. By manipulating the Wnt canonical signalling, cells metastasising to bone influence the phenotype of the metastatic osseous lesion [24,25,38-40].

Thus, aberrant activation of Wnt signalling pathway plays a pivotal role in the progression of the primary cancer and in determining whether the metastatic bone disease will be osteolytic or osteoblastic in type.

### Summary

The molecular pathways underlying EMT and stemness appear to be interlinked. Cancer stem cells and de-differentiated cancer cells express several transcription factors that under physiological conditions regulate embryonic development. During cancerization these transcription factors mediate increased survival and proliferation of cancer cells, and mediate cancer cell detachment, motility and migration, resulting in tumour invasion and metastasis.

## **Competing interests**

The authors declare that they have no competing interests.

## **Authors’ contribution**

The concept of this paper was devised by LF. LF, BK and JL contributed equally to the intellectual input of the paper. All authors read and approved the final manuscript.
